# Supplementary material for: Predicting and testing a gene network regulating seed germination in Arabidopsis
Source: PeerJ. 2025 Jul 7;13:e19599. doi: 10.7717/peerj.19599 (PMC12244130; doi:10.7717/peerj.19599)
Supplement: Supplemental Information 2 [file peerj-13-19599-s002.docx]

| Gene locus | Mutant line | Short mutant line name | Gene-specific Primer pair | Primer pair for T-DNA insertion |
| --- | --- | --- | --- | --- |
| At1g51170 | SALK_044862C exon | G3 | 741/742 | 741/LBa1 |
| At1g51170 | SALK_023783C | G4 | 741/742 | LBa1/742 |
| At3g13380 | CS800036 (SALK_006024 ) | G5 | 744/745 | 744/LBa1 |
| At3g13380 | SAIL_529_C11 / CS879501 | G | 744/745 | 744/LB3 |
| At2g23060 | SALK_013767C | G9 | 750/752 | 750/LBa1 |
| At2g23060 | SALK_075387C | G10 | 750/752 | 750/LBa1 |
| At1g78090 | CS457196 (GK-596G04) | Y1 | 753/754 | 753/768 |
| At1g78090 | CS65590 (SALK_037324) | Y2 | 755/756 | LBa1/756 |
| At4g35060 | NASC N441878 ( GK-437B10) | Y5 | 759/760 | 768/760 |
| At4g35060 | SALK_205622C | Y6 | 759/760 | LBa1/760 |

**Table S2.** Mutant lines and the corresponding PCR primers for genotyping
