# Supplementary material for: Predicting and testing a gene network regulating seed germination in Arabidopsis
Source: PeerJ. 2025 Jul 7;13:e19599. doi: 10.7717/peerj.19599 (PMC12244130; doi:10.7717/peerj.19599)
Supplement: Supplemental Information 3 [file peerj-13-19599-s003.docx]

| Primer | Sequence |
| --- | --- |
| 741 | GGATCGCTTACAGAGACTTGAA |
| 742 | GTTGGCTTCCGTCAGTTATCTA |
| 744 | GGTCTTACCGGAACCCTAAATC |
| 745 | GTGGAATCTCACCGGAGTAAAG |
| 750 | GGTTTAAGCTCGTGAAGGCTATG |
| 752 | GGTGCTTGACCTTTGTAATTGG |
| 753 | TAACAGAGATGGCCTGCATTAG |
| 754 | CCCTCCAGGCATCTACTAATTT |
| 755 | CCTACACGTCTCAGATCCTCTT |
| 756 | ACTTGTTGTTCTCCACCATAGC |
| 759 | CTCATACACGACTCACTGAAGG |
| 760 | CGAACACGCTATCGATGTCTAA |
| LBa1 (SALK LB primer)^*^ | TGGTTCACGTAGTGGGCCATCG |
| LB3 (SAIL LB primer)^*^ | TTCATAACCAATCTCGATACAC |
| 768 (GABI LB primer)^**^ | ATATTGACCATCATACTCATTGC |

**Table S3.** Polymerase chain reaction primers used for genotyping

^*^ http://signal.salk.edu/tdnaprimers.2.html

^**^ https://www.gabi-kat.de/
