# Supplementary material for: Predicting and testing a gene network regulating seed germination in Arabidopsis
Source: PeerJ. 2025 Jul 7;13:e19599. doi: 10.7717/peerj.19599 (PMC12244130; doi:10.7717/peerj.19599)
Supplement: Supplemental Information 4 [file peerj-13-19599-s004.docx]

| Mutant | T-DNA line | Short name | Insertion site | RT-PCR primer pair | RT-PCR product Size |
| --- | --- | --- | --- | --- | --- |
| *At1g51170* | SALK_044862C | G3R | Exon^*^ | 741/LBa1 | 606bp+310bp |
| *At1g51170* | SALK_023783C | G4 | Exon^*^ | 743/LBa1 | 73bp+310bp |
| *brl3/At3g13380* | CS800036/SALK_006024 | G5 | Exon^*^ | 744/LBa1 | 187bp+310bp |
| *brl3/At3g13380* | CS879501/SAIL_529_C11 | G6 | Exon^*^ | 744/LB3 | 144bp+102bp |
| *At2g23060* | SALK_013767C | G9 | 3rd exon | 750/LBa1 | 758bp+310bp |
| *At2g23060* | SALK_075387C | G10 | 3rd exon | 750/LBa1 | 363bp+310bp |
| *At1g78090* | CS457196/GK-596G04 | Y1 | 3rd exon | 777/768 | 346bp+68bp |
| *At1g78090* | CS65590/SALK_037324 | Y2 | 10th exon | 756/LBa1 | 244bp+310bp |
| *hmp39*/*At4g35060* | N441878/GK-437B10 | Y5 | 1st exon | 790/768 | 376bp+68bp |
| *hmp39*/*At4g35060* | SALK_205622C | Y6 | 3’-UTR | 791/LBa1 | 441bp+310bp |

**Table S4.** Locations of the T-DNA insertions and the expected RT-PCR products in the

^*^The coding region of the gene has only one exon and no intron.
