# Supplementary material for: Predicting and testing a gene network regulating seed germination in Arabidopsis
Source: PeerJ. 2025 Jul 7;13:e19599. doi: 10.7717/peerj.19599 (PMC12244130; doi:10.7717/peerj.19599)
Supplement: Supplemental Information 5 — G3R and G4: mutants of At1g51170; G5 and G6R: brl3 mutants; G9 and G10R: mutants of At2g23060; Y1 and Y2: mutants of At1g78090; and Y5 and Y6: hmp39 mutants. Primer pairs containing either LBa1 or 768 denote the T-DNA insertions. A homozygous mutant had the insertion band but not the Col-0 (wild-type) band. [file peerj-13-19599-s005.pdf]

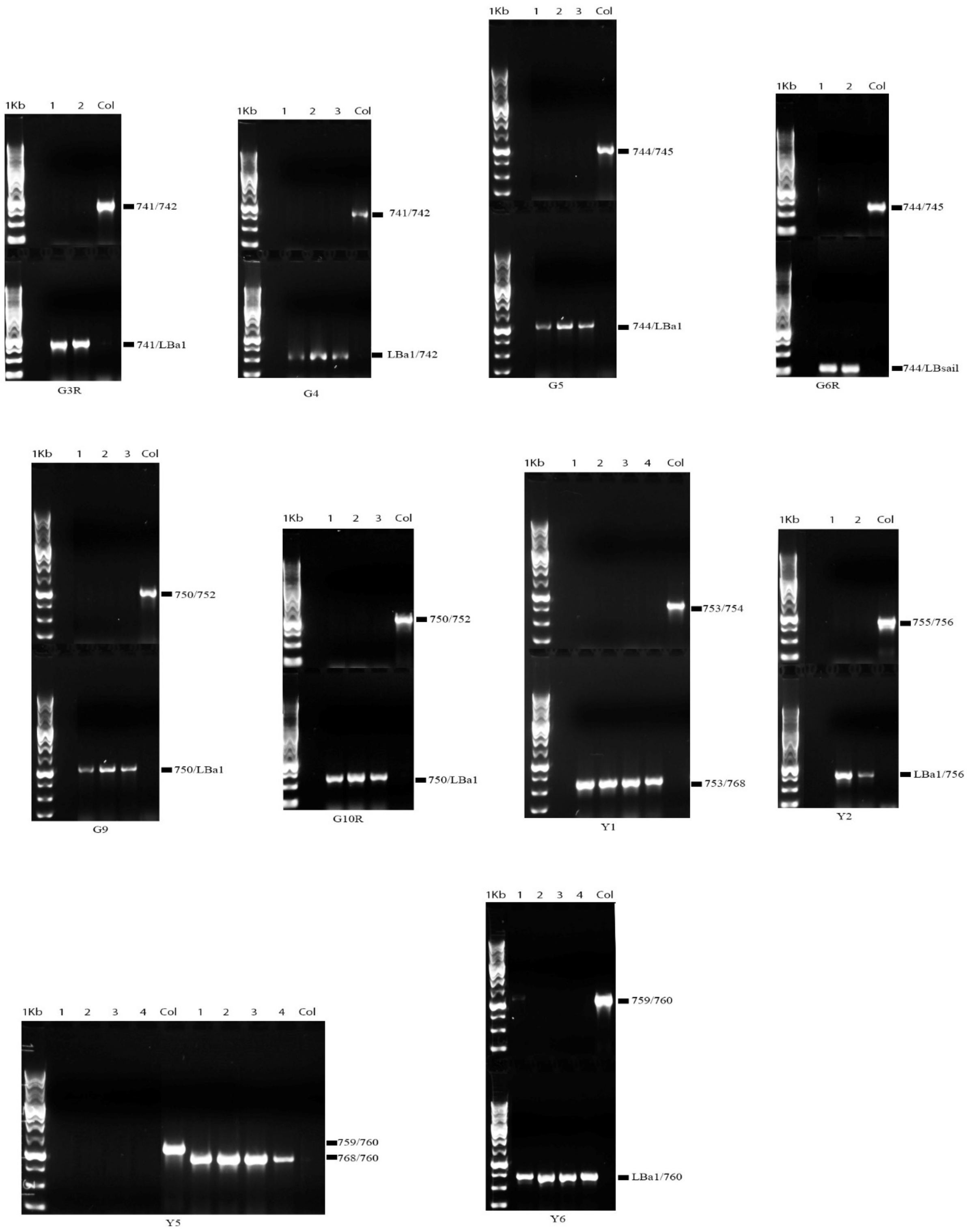

Fig. S1. Identification of homozygous mutants for seed germination tests. G3R and G4: mutants of At1g51170; G5 and G6R: *brl3* mutants; G9 and G10R: mutants of At2g23060; Y1 and Y2: mutants of At1g78090; and Y5 and Y6: *hmp39* mutants. Primer pairs containing either LBa1 or 768 denote the T-DNA insertions. A homozygous mutant had the insertion band but not the Col-0 (wild-type) band.
