# Supplementary material for: Predicting and testing a gene network regulating seed germination in Arabidopsis
Source: PeerJ. 2025 Jul 7;13:e19599. doi: 10.7717/peerj.19599 (PMC12244130; doi:10.7717/peerj.19599)
Supplement: Supplemental Information 6 — This was created in Cytoscape_3.9.1. and was used as the basis for Fig. 6. Edges ending with an arrow: upregulation; edges ending with a horizontal or vertical bar: downregulation. [file peerj-13-19599-s006.pdf]

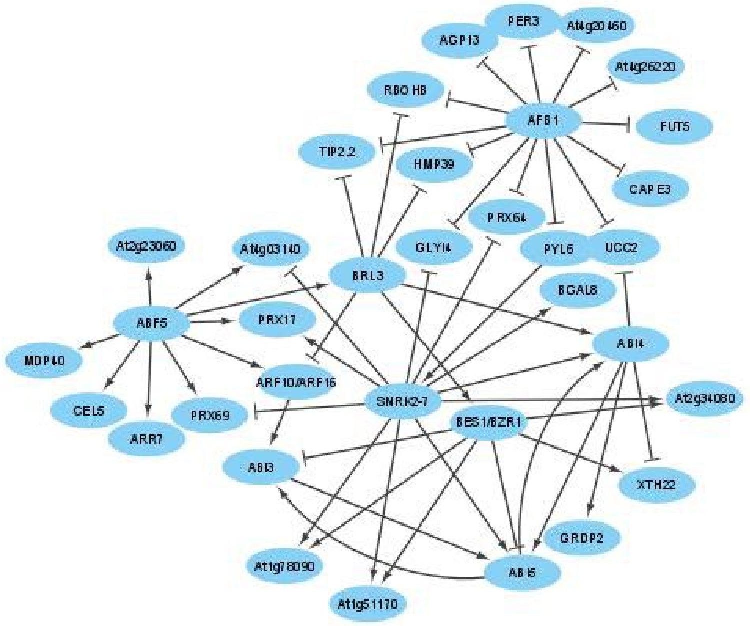

Fig. S2. A gene network for controlling seed germination in Arabidopsis. It was created in Cytoscape_3.9.1. and was used as the basis for Fig. 6. Edges ending with an arrow: upregulation; edges ending with a horizontal or vertical bar: downregulation.
